# Understanding cell behavior in cultivation processes - A metabolic approach

**DOI:** 10.1186/1753-6561-7-S6-P90

**Published:** 2013-12-04

**Authors:** Jonas Aretz, Tobias Thüte, Sebastian Scholz, Klaudia Kersting, Thomas Noll, Heino Büntemeyer

**Affiliations:** 1 Institute of Cell Culture Technology, Bielefeld University, Bielefeld, Germany; 2 Center for Biotechnology (CeBiTec), Bielefeld University, Bielefeld, Germany

## Background

During cultivation cells undergo a tremendous change in their metabolism when shifting from one state to another or when parameters are changed. To understand the changes in intracellular metabolite concentrations and their impact on cell performance we used a systematic approach. By employing the chemostat mode at different steady state conditions we investigated the alterations of the concentrations of key metabolites during cultivations of a human production cell line.

## Methods

Chemostat cultivations were performed with the AGE1.hn AAT cell line (Probiogen AG, Berlin, Germany) and TC-42 medium (Teutocell AG, Bielefeld, Germany) in a fully controlled 2 litre benchtop bioreactor (Sartorius, Göttingen,Germany). Different dilution rates of 0.24 d^-1^, 0.33 d^-1^, and 0.40 d^-1 ^and pH values of pH 6.9, pH 7.15, and pH 7.3 were performed using the same bioreactor setup. For stopping the cell metabolism an established fast filtration method [1] was used for rapid quenching. Metabolites were extracted from cells using liquid/liquid extraction. Extracts were analyzed by using hydrophilic interaction chromatography (HILIC) and ESI-MS/MS mass spectometry. Extracellular amino acids and pyruvate were analyzed by pre-column derivatization and RP-HPLC [2], glucose and lactate using a YSI 2700 bioanalyser.

## Results

**Figure 1:**
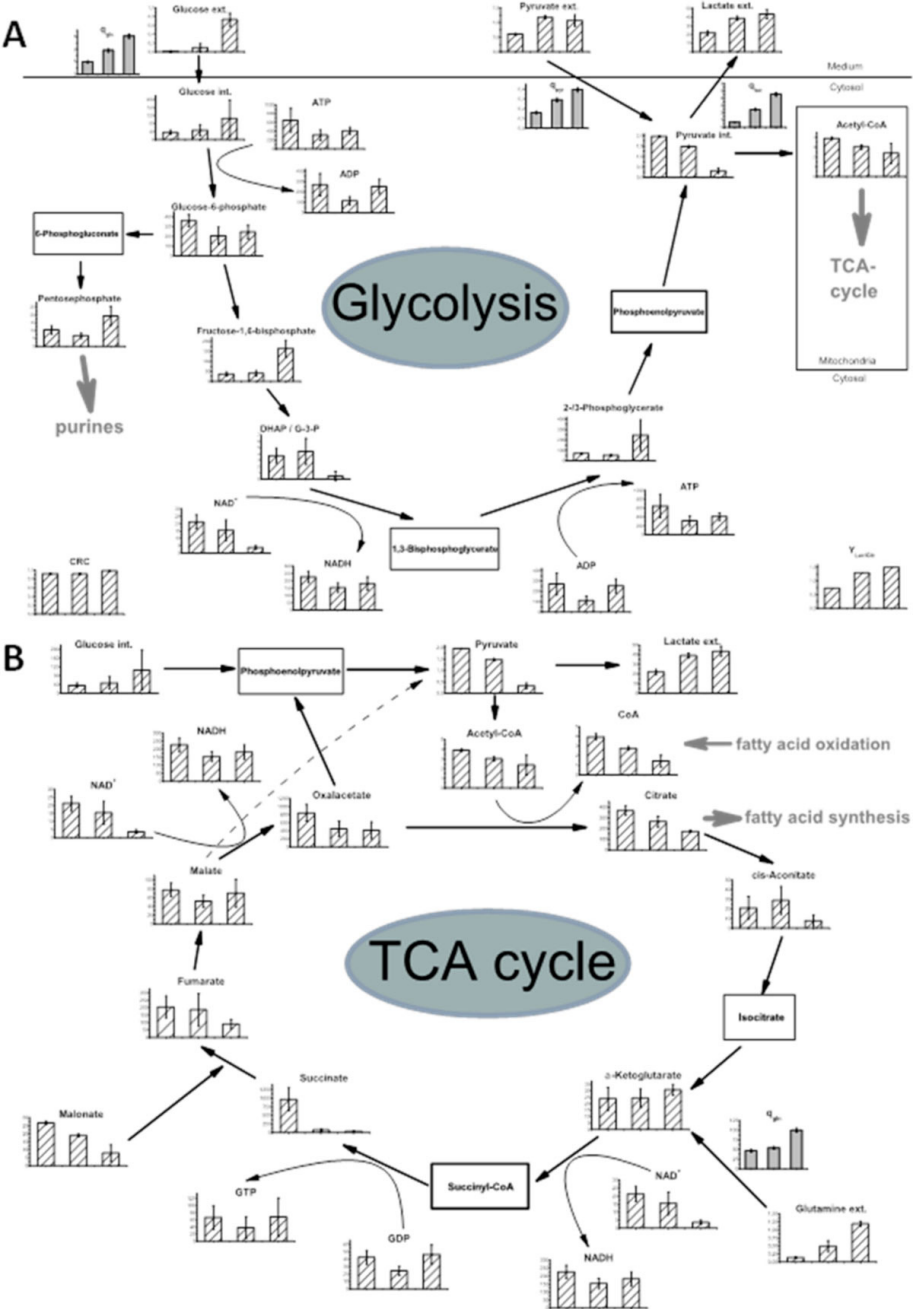
**Metabolite pool sizes in Glycolysis (A) and TCA (B) at different dilution rates. The metabolism at the three different dilution rates 0,24 d^-1 ^(left), 0,33 d^-1 ^(middle), 0,4 d^-1 ^(right) is shown**. Specific rates are illustrated with filled bars and given in nmol cell^-1 ^d^-1^. Stripped bars illustrate pool sizes which are given in mM (extracellular) and μM (intracellular), respectively.

The comparative analysis of the three steady state dilution rates shows the great impact of changing extracellular conditions on the intracellular metabolite pools which may also lead to an altered productivity. For example, as been shown in Figure 1A the specific pyruvate consumption rate, qPyr, as well as the intracellular pyruvate pools decrease with increasing dilution rates, while qGlc and qGln increase at the same time. While some metabolite pools show great differences between different dilution rates others remain more or less constant. A malonate inhibition of the TCA cycle (Figure 1B) appears mainly at low dilution rates, which might be an effect of glucose and / or glutamine limitation at those steady states.

Although qGlc, qPyr as well as qGln decrease with increasing pH values (data not shown), the intracellular TCA pools remain constant due to a catabolism of further amino acids (Table 1). This may have led to a lower waste of ammonia, lactate and glycine at higher pH values.

**Table 1:** Correlation of specific rates q_xxx _with the adjusted pH values during steady state

	pH 6,9	pH 7,15	pH 7,3
q_NH3_	430 ± 27	243 ± 9	207 ± 19
q_Lac_	4751 ± 298	3766 ± 143	3548 ± 325
q_Glc_	- 3660 ± 230	- 3302 ± 126	- 3301 ± 302
q_Pyr_	- 155 ± 10	- 121 ± 5	-84 ± 8
q_Gln_	- 527 ± 33	- 488 ± 19	- 484 ± 44
q_Asp_	- 63 ± 4	- 123 ± 5	-153 ± 14
q_Glu_	66 ± 4	29 ± 1	- 16 ±2
q_Asn_	- 17 ±1	- 42 ± 2	-45 ± 4
q_Ser_	-91 ± 6	-198 ± 8	- 191 ± 17
q_His_	- 13 ± 1	- 23 ± 1	-5 ± 1
q_Gly_	32 ± 2	9 ± 0	7 ± 1
q_Thr_	- 26 ± 2	61 ± 2	67 ± 6
q_Arg_	- 39 ±2	- 97 ± 4	- 109 ± 10
q_Ala_	101 ± 6	48 ± 2	99 ± 9
q_Tyr_	- 10 ± 1	-29 ± 1	29 ± 2
q_Met_	-20 ± 1	-39 ± 2	- 40 ± 4
q_Val_	-37 ± 2	-79 ± 3	- 88 ± 8
q_Trp_	- 5 ± 0	- 8 ± 0	- 9 ± 1
q_Phe_	- 10 ± 1	-36 ± 1	-36 ± 3
q_Ile_	- 35 ± 2	- 68 ± 3	- 72 ± 7
q_Leu_	- 63 ± 4	-111 ± 4	-122 ± 11
q_Lys_	- 21 ± 1	- 89 ± 3	- 100 ± 9

The specific rates are given in pmol cell^-1 ^d^-1^. (Negative values indicate consumed metabolites.)

The analysis of the intracellular nucleotide pools show that while the concentrations of almost all nucleotides dropped with increasing dilution rates, they were more or less stable at changing pH values (data not shown).

## Conclusions

Although more data have to be raised to get a comprehensive insight into cell metabolism it could be shown that chemostat cultures performed at steady state conditions are a valuable tool for investigating cell behaviour on an intracellular basis. A much better data stability can be obtained than in batch or fed-batch cultures.
